# Supplementary material for: Genetic background and immune response in paracoccidioidomycosis: A systematic review and meta-analysis of single nucleotide variants
Source: PLoS Negl Trop Dis. 2026 Mar 19;20(3):e0014110. doi: 10.1371/journal.pntd.0014110 (PMC13001940; doi:10.1371/journal.pntd.0014110)
Supplement: Attachment — Submitted filename: respostas_editor.docx [file pntd.0014110.s006.docx]

1) Please provide an Author Summary. This should appear in your manuscript between the Abstract (if applicable) and the Introduction, and should be 150-200 words long. The aim should be to make your findings accessible to a wide audience that includes both scientists and non-scientists. Sample summaries can be found on our website under Submission Guidelines:

*Paracoccidioidomycosis is a fungal disease that affects mainly rural workers in Latin America, especially in Brazil, and remains largely neglected despite its social and health impact. People exposed to the fungus can develop different forms of the disease, from mild to severe, suggesting that individual biological factors play an important role. In this study, we asked whether genetic differences between people help explain who becomes ill and how severe the disease can be. We systematically reviewed published studies that investigated genetic variants in immune-related genes and their association with paracoccidioidomycosis. We also combined available data using meta-analysis when the same genetic variants were evaluated in more than one study. Although some studies suggested that variants in genes related to immune regulation and vitamin D signaling might influence susceptibility to the disease, these findings were not confirmed when the data were analyzed together. Our results show that current evidence linking human genetic variation to paracoccidioidomycosis is limited and inconsistent. Most studies included few participants and differed in their design, populations, and selection of control groups. We conclude that larger, better-designed studies are needed to clarify whether genetic factors truly influence the risk and clinical outcomes of this neglected fungal disease.*

2) We noticed that you used the phrase 'unpublished data' in the manuscript. We do not allow these references, as the PLOS data access policy requires that all data be either published with the manuscript or made available in a publicly accessible database. Please amend the supplementary material to include the referenced data or remove the references.

*In this study, we contacted the original authors to obtain additional information that was not reported in the published articles, such as collection site and sample size.*

*Page 10, lines 228-230: “We approached study authors to obtain additional data that were not available in the published articles, such as collection site and sample size.”*

*As requested by the journal, all information retrieved through direct communication with the authors has now been fully included in S3 Table, ensuring transparency and public availability of these data. This has also been explicitly stated in the revised manuscript (page 16, lines 319–321). We have revised the text to clarify that these data are no longer unpublished and are available as part of the supplementary material.*

3) We have noticed that you have uploaded Supporting Information files, but you have not included a complete list of legends. Please add a full list of legends for your Supporting Information files after the references list.

*Thank you for your comment regarding the Supporting Information legends. We would like to clarify that a complete list of legends for all Supporting Information files is already included at the end of the manuscript, on pages 46–47, lines 1053–1082.*

4) Please provide a detailed Financial Disclosure statement. This is published with the article. It must therefore be completed in full sentences and contain the exact wording you wish to be published.

*This study was financed in part by the Coordenação de Aperfeiçoamento de Pessoal de Nível Superior - Brasil (CAPES) - Finance Code 001 (SSC and WSF) and Universidade Federal de Mato Grosso do Sul- Brasil (UFMS) - Finance Code 001. The funders had no role in study design, data collection and analysis, decision to publish, or preparation of the manuscript*
