# Supplementary material for: Genetic background and immune response in paracoccidioidomycosis: A systematic review and meta-analysis of single nucleotide variants
Source: PLoS Negl Trop Dis. 2026 Mar 19;20(3):e0014110. doi: 10.1371/journal.pntd.0014110 (PMC13001940; doi:10.1371/journal.pntd.0014110)
Supplement: Attachment — Submitted filename: Response to Reviewers.docx [file pntd.0014110.s007.docx]

**Comment 1 – Figure resubmission: *While revising your submission, we strongly recommend that you use PLOS’s NAAS tool (***[***https://ngplosjournals.pagemajik.ai/artanalysis***](https://ngplosjournals.pagemajik.ai/artanalysis)***) to test your figure files. NAAS can convert your figure files to the TIFF file type and meet basic requirements (such as print size, resolution), or provide you with a report on issues that do not meet our requirements and that NAAS cannot fix. After uploading your figures to PLOS’s NAAS tool, NAAS will process the files provided and display the results in the “Uploaded Files” section. If the uploaded figures meet our requirements (or NAAS is able to fix the files), the figure will be marked as “fixed”. If NAAS is unable to fix the files, a red “failed” label will appear. When NAAS has confirmed that the figure files meet our requirements, please download the processed files and include them when submitting your revised manuscript.***

**Authors:** We acknowledge and appreciate this recommendation. All figure files included in the revised submission were processed using the PLOS NAAS tool to ensure full compliance with the journal’s technical requirements regarding file format, resolution, and print quality. The NAAS-processed figure files were downloaded directly from the platform after successful validation and are included in the current resubmission.

**Comment 2 – Reproducibility: *To enhance the reproducibility of your results, we recommend that authors of applicable studies deposit laboratory protocols in protocols.io, where a protocol can be assigned its own identifier (DOI) such that it can be cited independently in the future. Additionally, PLOS ONE offers an option to publish peer-reviewed clinical study protocols.***

**Authors:** We thank the Editors for this recommendation. The present study is a systematic review and meta-analysis and does not involve original laboratory experiments or clinical intervention protocols. Therefore, there are no laboratory or clinical protocols applicable for deposition in protocols.io. Nevertheless, we have ensured transparency and reproducibility by comprehensively reporting our search strategy, eligibility criteria, data extraction procedures, risk-of-bias assessment, statistical analyses, and meta-analytic methods, in accordance with PRISMA and Cochrane guidelines. In addition, the study was prospectively registered in a public registry (PROSPERO, registration number CRD42025646417).

**Reviewers' Responses**

**Reviewer #1. *The manuscript by Coelho et al., addresses an important topic: the genetic basis of susceptibility to paracoccidioidomycosis (PCM), a neglected tropical disease endemic to Latin America. The review and meta-analysis are relevant. However, the study faces methodological limitations that affect the robustness of its conclusions***.

**Authors.** We thank the reviewer for the careful evaluation of our manuscript and for recognizing the relevance of addressing the genetic basis of susceptibility to paracoccidioidomycosis. We acknowledge the methodological limitations highlighted and agree that they may affect the robustness of the conclusions. In response, the manuscript was substantially restructured, with a clearer distinction between levels of evidence, a more cautious interpretation of the findings, and explicit discussion of the limitations inherent to the available studies, as detailed in the responses below.

**Reviewer #1.** ***The meta-analysis includes only two studies per SNV, which severely limits statistical power. This should be emphasized more clearly in the abstract and discussion.***

**Authors**. We thank the reviewer for this important observation. We fully agree that the inclusion of only two studies per SNV substantially limits the statistical power of the meta-analyses and constrains the interpretation of the pooled estimates. This limitation is not methodological in nature, but rather reflects the current state of the literature, in which few primary studies have evaluated the same genetic variants using comparable designs.

In response to this comment, we have revised both the Abstract (Page 2, lines 45-48) and the Discussion (Pages 23-24, lines 503-514) to explicitly emphasize the low statistical power and the exploratory nature of the meta-analytical findings. We now clarify that the absence of significant associations in the meta-analyses should not be interpreted as evidence against a role of host genetic variability in PCM susceptibility, but rather as a consequence of limited sample sizes, lack of replication, and heterogeneity across studies.

We also highlight that a central contribution of this systematic review is precisely to organize the existing evidence critically, prevent overinterpretation of isolated positive findings, and objectively expose gaps in the literature, thereby underscoring the need for larger, well-powered, and methodologically harmonized primary studies in this field.

**Reviewer #1.** ***Funnel plot asymmetry tests were omitted due to insufficient studies, this limitation should be stated in the methods and discussion.***

**Authors**. We thank the reviewer for this comment and fully agree with the observation. The omission of funnel plot asymmetry tests was a deliberate and methodologically appropriate decision, rather than an oversight. According to established guidelines, including those from the Cochrane Handbook, tests for funnel plot asymmetry are not recommended when meta-analyses include a small number of studies, as they lack sufficient power to provide meaningful or reliable results.

In response to this comment, we have revised both the Methods (Page 11, lines 243-248) and Discussion (Pages 23-24, lines 503-514) sections to explicitly state that, given the inclusion of only two studies per SNV, funnel plot asymmetry tests were not performed because they would not be informative or statistically appropriate.

**Reviewer #1.** ***Only one study applied multiple testing correction. The absence of FDR or Bonferroni adjustments in most studies increases the risk of false positives. This limitation should be more prominently addressed.***

**Authors**. We thank the reviewer for this important and well-founded observation. We fully agree that the lack of multiple testing correction in most primary studies represents a relevant limitation of the available evidence and increases the risk of false-positive associations. As noted by the reviewer, only one of the included studies applied formal correction for multiple comparisons, such as FDR or Bonferroni adjustments. This issue reflects a structural limitation of the current literature on genetic susceptibility to PCM rather than a methodological shortcoming of the present review. While the absence of multiple testing correction does not automatically invalidate the original findings, it clearly requires cautious interpretation, particularly in studies with small sample sizes and multiple SNVs evaluated simultaneously. Therefore, we have revised the Discussion (Pages 26-27, lines 558-589) to more prominently emphasize this limitation and to explicitly acknowledge that some reported associations may represent spurious findings driven by insufficient statistical control. We also highlight this issue as a key methodological gap that should be addressed in future studies through appropriate correction for multiple comparisons, larger sample sizes, and replication strategies.

**Reviewer #1.** ***The discussion is somewhat repetitive. It also should separate methodological limitations from biological interpretation.***

**Authors**. We carefully revised the Discussion to reduce redundancy, improve textual flow, and strengthen its overall structure. Specifically, we rewrote the section spanning on Pages 26-27, lines 558-589, to more clearly separate methodological considerations, such as limitations related to exposure assessment, potential sources of bias, and statistical power—from the biological interpretation of the findings.

**Reviewer #1.** ***Figure captions must be improved to make them self-explanatory.***

**Authors**. As requested, we have revised all figure captions to make them more self-explanatory, providing clearer descriptions of the analyses performed and the information conveyed by each figure.

**Reviewer #2**. ***This manuscript by Coelho and colleagues addresses an important and underexplored topic. It looks at how host genetic differences affect susceptibility and clinical outcomes in paracoccidioidomycosis (PCM), which is a neglected disease relevant to public health in Latin America. The authors conducted a systematic review and reanalysis of published studies that focus on single nucleotide variants (SNVs) in immune-related genes and carried out a limited meta-analysis. Their effort to critically reassess published data, including Hardy-Weinberg equilibrium (HWE), inheritance models, and effect sizes, is commendable and meets the standards expected by this journal. However, despite its strengths, the manuscript has some problems that significantly limit its impact and may bias the conclusions. The main issues are discussed below.***

**Authors**. We sincerely thank the Reviewer for the careful and insightful assessment of our manuscript and for acknowledging the relevance of this underexplored topic, as well as the methodological rigor applied in the reanalysis of published data. We fully recognize that, despite these strengths, several limitations may restrict the impact of the study and introduce potential bias in the conclusions. In response, we undertook a substantial revision of the manuscript, restructuring key sections, refining the analytical interpretation, and explicitly addressing the methodological constraints and sources of bias identified, as discussed in detail in the points below.

**Reviewer #2. *A central limitation of the manuscript is the assumption that PCM susceptibility and severity are driven mainly by host genetic and immunological factors. While the introduction and discussion emphasize host immune responses, such as the Th1/Th2 balance, cytokine polymorphisms, PRRs, and VDR signaling, they largely overlook the role of the fungal pathogen itself. PCM is defined by host-pathogen interaction. There are well-documented differences in virulence, immunomodulatory capacity, thermotolerance, antigen expression, and cell wall composition among Paracoccidioides species and strains, such as P. brasiliensis and P. lutzii. These differences have significant effects on disease outcomes. The manuscript fails to adequately discuss how fungal genetic diversity, virulence factors, or strain-specific traits may influence host genetic associations. This omission risks presenting an oversimplified and potentially misleading view that PCM is “primarily a host-driven disease.” In reality, disease development stems from a dynamic interaction between host susceptibility and fungal pathogenicity. Additionally, it should be noted that the studies cited in references 7, 8, 9, 10, and 11 exclusively used the P. brasiliensis Pb18 isolate. Prolonged in vitro cultivation of pathogenic fungi is known to lead to significant changes in the expression of virulence-related components, often resulting in attenuated phenotypes. This laboratory adaptation can affect cell wall composition, secretion of enzymes, host-pathogen interactions, and stress response pathways. Therefore, relying on a single isolate that may have undergone changes due to long-term culture presents an additional limitation of these studies. This should be considered when interpreting conclusions related to fungal virulence and host-pathogen interactions. The authors should acknowledge this limitation and frame their conclusions within a host-pathogen interaction model instead of a host-centric view.***

**Authors**. We thank the reviewer for this thoughtful and important comment. We fully agree that paracoccidioidomycosis (PCM) is a complex disease resulting from a dynamic interaction between host susceptibility and fungal pathogenicity. In response to this comment, we have revised the manuscript to better frame PCM within a host–pathogen interaction model rather than a host-centric view. Specifically, we expanded the Introduction (pages 4-6, lines 70-123) and Discussion sections (pages 31, lines 688-704).

**Reviewer #2**. ***Though the manuscript correctly highlights associations involving IL10 and VDR SNVs, the biological interpretation remains shallow. Most of the variants mentioned are found in promoter, intronic, or untranslated regions, yet there is little discussion regarding whether these SNVs have any functional impact, such as altered cytokine production, receptor expression, or signaling efficiency in PCM or related fungal infections.***

**Authors**. We thank the reviewer for this insightful comment. As requested, we have revised the Discussion section (pages 22–24, lines 457–514) to expand the functional interpretation of the main IL10 and VDR SNVs identified in this study. The revised text now explicitly addresses how promoter and regulatory-region variants in IL10 may modulate cytokine expression levels and immune regulation, and how VDR polymorphisms located in intronic or untranslated regions may affect receptor expression, vitamin D signaling efficiency, and host immune responses.

**Reviewer #2**. ***Another significant weakness is the inconsistency in defining “exposure” and “controls” across studies. Only two studies included controls with confirmed exposure to Paracoccidioides, while the others depended on apparently healthy individuals whose exposure status was unknown. Given the high prevalence of subclinical infection in endemic areas, this poses a critical confounding factor. The manuscript notes that none of the significant associations were convincingly tied to clinical form or disease severity, but it doesn’t integrate this finding into its conclusions effectively. As a result, the conclusions about “susceptibility” are vague and may be confused with exposure, infection, and progression.***

**Authors**. We fully agree that there is substantial heterogeneity across the included studies regarding the definition of “exposure” and the composition of control groups. As correctly pointed out by the reviewer, this limitation cannot be corrected at the level of a systematic review; however, it can and should be critically highlighted. Accordingly, we revised the manuscript (pages 29-30, lines 541-678) to more explicitly emphasize this methodological heterogeneity as a central limitation of the existing literature. In addition, we refined the Conclusions section to avoid overinterpretation of genetic “susceptibility”. We now explicitly state that the available evidence does not allow susceptibility to be clearly disentangled from exposure or infection status, nor does it support consistent associations between genetic variants and clinical form or disease severity. The absence of robust and reproducible genetic associations with disease progression or severity is now directly integrated into the conclusions, reinforcing that current findings should be interpreted with caution.

**Reviewer #2. *The introduction focuses heavily on immunology and could benefit from a brief section that recognizes fungal diversity and virulence as important factors in PCM.***

**Authors**. We thank the reviewer for the comment and, as requested, we have revised the manuscript to better frame PCM within a complex host–pathogen interaction model rather than a host-centric view. Specifically, we expanded the Introduction (pages 4-6, lines 70-123) and Discussion sections (pages 31, lines 688-704).

**Reviewer #2. *Some repetition occurs in the Discussion concerning HWE and multiple testing corrections; this could be streamlined***.

**Authors**. We carefully revised the Discussion to reduce redundancy, improve textual flow, and strengthen its overall structure. Specifically, we rewrote the section spanning on Pages 26-27, lines 558-689, to more clearly separate methodological considerations, such as limitations related to exposure assessment, potential sources of bias, and statistical power—from the biological interpretation of the findings.

**Reviewer #2.** ***The authors might consider including a reformulated version of Supplementary Table 1S, retaining its main points of the table, in the main text, as this could improve clarity and strengthen the presentation of the results.***

**Authors**. We thank the reviewer for this suggestion. In response, we reformulated Supplementary Table S1 to retain its key information and included this revised version in the main text. This modification was made to improve clarity and strengthen the presentation of the results, as suggested.
